# A Case of Recurrent Esophageal Cancer Treated with Concurrent Chemoradiation Therapy in Pregnancy

**DOI:** 10.1155/2018/1280582

**Published:** 2018-12-03

**Authors:** Kaori Yamada, Yoshitsugu Chigusa, Motoo Nomura, Katsuyuki Sakanaka, Mitsuhiro Nakamura, Shinsuke Yano, Shigeru Tsunoda, Eiji Kondoh, Masaki Mandai

**Affiliations:** ^1^Department of Gynecology and Obstetrics, Kyoto University, 54 Shogoin Kawahara-cho, Sakyo-ku, Kyoto 606-8507, Japan; ^2^Department of Therapeutic Oncology, Kyoto University, 54 Shogoin Kawahara-cho, Sakyo-ku, Kyoto 606-8507, Japan; ^3^Department of Radiation Oncology and Image-Applied Therapy, Kyoto University, 54 Shogoin Kawahara-cho, Sakyo-ku, Kyoto 606-8507, Japan; ^4^Division of Medical Physics, Department of Information Technology and Medical Engineering, Human Health Sciences, Graduate School of Medicine, Kyoto University, 54 Shogoin Kawahara-cho, Sakyo-ku, Kyoto 606-8507, Japan; ^5^Division of Clinical Radiology Service, Kyoto University Hospital, 54 Shogoin Kawahara-cho, Sakyo-ku, Kyoto 606-8507, Japan; ^6^Department of Surgery, Kyoto University, 54 Shogoin Kawahara-cho, Sakyo-ku, Kyoto 606-8507, Japan

## Abstract

Esophageal cancer rarely coincides with pregnancy, and only five cases have been reported thus far. The management of esophageal cancer during pregnancy is extremely challenging due to its aggressive nature. We herein report a case of recurrent esophageal cancer in pregnancy. A 41-year-old multigravida with a history of esophageal squamous cell cancer treated with esophagectomy and perioperative chemotherapy was diagnosed with local recurrent carcinoma of the residual esophagus at 16 weeks of gestation. The patient strongly desired to continue the pregnancy, and concurrent chemoradiation therapy (CRT) consisting of 50.4 Gy of radiation, cisplatin, and 5-fluorouracil was carried out from 19 weeks of gestation. CRT was dramatically effective, and the recurrent lesion disappeared. At 38 weeks of gestation, she underwent cesarean section and delivered a healthy female baby. Both maternal and fetal courses were satisfactory, and the patient has been free of disease for 12 months. This is the first case of recurrent esophageal cancer in pregnancy in which CRT was completed without reducing treatment intensity and led to a complete response. Nevertheless, little is known regarding the safety and possible adverse effects of CRT on the fetus. Therefore, deliberate selection of patients and long-term follow-up of the child are necessary.

## 1. Introduction

With increasing maternal age and improvements in diagnostic techniques, the incidence of cancer in pregnancy has risen to one in 1,000 pregnancies [1], and it is not necessarily a rare entity. However, esophageal cancer complicated with pregnancy is extremely uncommon. Thus, its management strategy has not yet been discussed thoroughly. Here, we describe a case of recurrent esophageal cancer treated successfully with concurrent chemoradiation therapy (CRT) during pregnancy. Further, we also review previously reported cases of esophageal cancer in pregnancy.

## 2. Case Report

**Figure 1:**
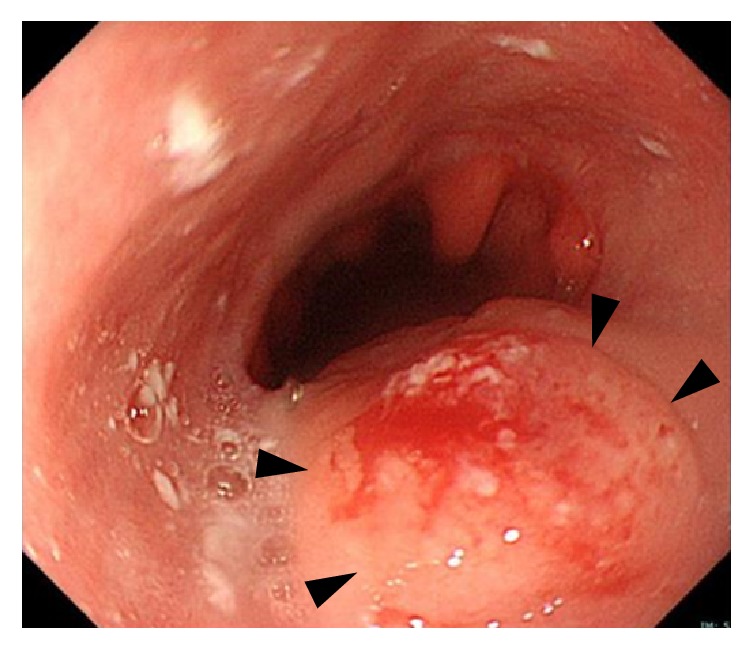
The gastrointestinal endoscopic finding at 16 weeks of gestation. Arrowheads indicate the recurrent lesion of esophageal cancer in the residual esophagus.

A 41-year-old woman, gravida 2, para 1, was diagnosed with esophageal cancer relapse at 16 weeks of gestation. At the age of 40, before this spontaneous conception, she underwent preoperative chemotherapy, minimally invasive esophagectomy, and postoperative chemotherapy for esophageal squamous cell cancer, cStageII, pStageIV. Since the postoperative chemotherapy, she had been amenorrhoeic. Ten months after the operation, contrast enhanced computed tomography (CT) of the neck, chest, and abdominal to pelvis region was performed to investigate recurrence or metastasis, and it showed a pregnant uterus. She visited our obstetric clinic and was confirmed to be at 8 weeks of gestation. Because the estimated fetal exposure dose of the CT examination was less than 8 mGy, she wished to continue the pregnancy. At 16 weeks of gestation, the scheduled gastrointestinal endoscopy and biopsy revealed relapse of esophageal cancer in the residual esophagus (Figure 1). The fluorine-18 fluorodeoxyglucose (18F-FDG) uptake of the local recurrence in the residual esophagus was elevated according to positron emission tomography (PET)/CT, and no metastatic lesion was detected. A multidisciplinary treatment team consisting of medical oncologists, radiation oncologists, surgeons, and obstetricians recommended that the patient terminate the pregnancy and receive CRT, which was the standard treatment for localized recurrent disease [2, 3]. However, the patient and her husband strongly desired to continue the pregnancy.

**Figure 2:**
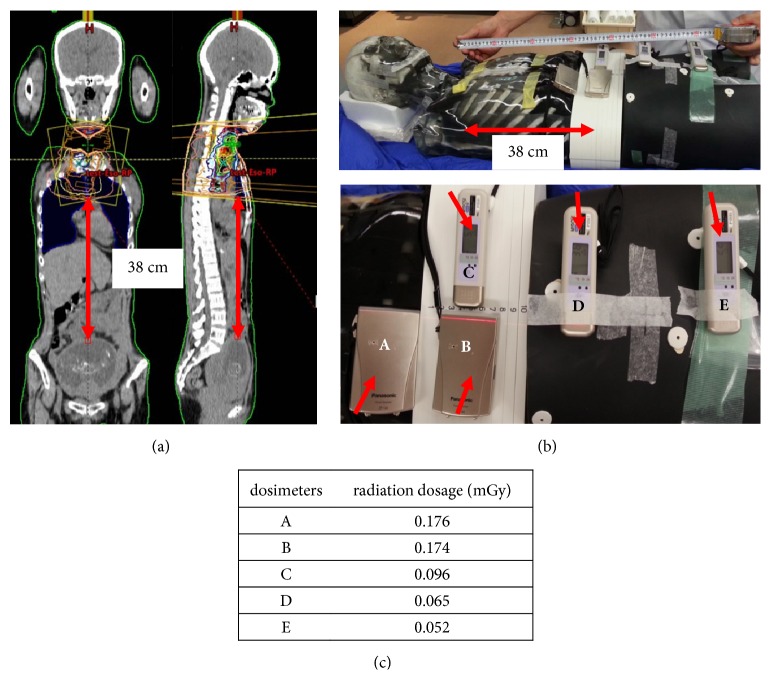
The simulation study of radiotherapy using a phantom. (a) The irradiation plan was created based on the recurrent lesion. The distance between the inferior margin of irradiation and the uterine fundus was 38 cm. (b) Five dosimeters (arrows) were put on the phantom's abdomen. (c) The radiation dosage measured by five dosimeters in one irradiation fraction.

To examine the safety and feasibility of radiotherapy for this patient, the fetal dose was estimated by a simulation study before CRT. We created the irradiation plan for the patient (Figure 2(a)) and delivered doses to the phantom (Figure 2(b)). According to the radiation dosage measured by five dosimeters, which ranged from 0.052 to 0.176 mGy in one irradiation fraction (Figure 2(c)), the fetal dose was estimated as 1.56 to 5.28 mGy, even after 60 Gy in 30 fractions of radiotherapy. The anticancer drugs, which would be administered together with the radiation, were cisplatin (CDDP) and 5-fluorouracil (5-FU). Taking these results, we concluded that CRT during pregnancy in this case would be acceptable and, after receiving written informed consent, started the treatment at 19 weeks of gestation.

**Figure 3:**
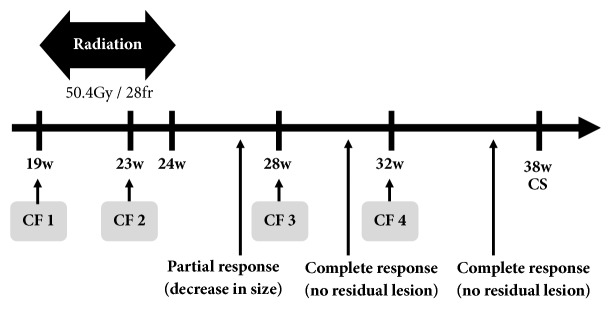
The time course of treatment. The horizontal axis indicates gestational weeks. CF, cisplatin 60 mg/m^2^, 5-fluorouracil 750 mg/m^2^; CS, cesarean section.

The CRT consisted of 50.4 Gy in 28 fractions of radiation and four courses of chemotherapy (CDDP 60 mg/m^2^ on day 1, 5-FU 750 mg/m^2^ on days 1 to 4).  Figure 3 shows the course of treatment over time. As supportive therapy, palonosetron and dexamethasone were administered. To assess the actual fetal dose of radiotherapy, we put dosimeters on the patient's abdomen at every irradiation session. Dosimeters showed that the estimated fetal dose ranged from 0.08 to 0.34 mGy in one irradiation fraction. Consequently, the multidisciplinary team confirmed the safety and feasibility of radiotherapy for this pregnant patient and fetus and completed the planned radiotherapy. After chemoradiotherapy, at 27 weeks of gestation, the recurrent lesion was decreased in size. Furthermore, gastrointestinal endoscopy at 31 weeks did not detect the recurrent lesion after the third course of chemotherapy. During this treatment, the myelosuppression was mild; grade 2 anemia, grade 1 neutrophil count decreased, and grade 1 platelet count decreased, according to Common Terminology Criteria for Adverse Events (CTCAE) Version 5.0. The patient had also pharyngitis, anorexia, and vomiting. Regarding body habitus, her height was 162 cm, and her weight was 51 kg before the esophagectomy. Then she had lost 6 kg and her body mass index was 17.5 by the time she got pregnant. Her weight gain was only 3.9 kg during pregnancy because of impaired oral intake due to gastric tube reconstruction as well as adverse effect of CRT such as anorexia. The fetal estimated body weight fluctuated between the 10th and 20th percentile. Fetal well-being was monitored using ultrasound and nonstress fetal heart rate testing, and no abnormal findings were detected. At 38 weeks and 3 days of gestation, she underwent cesarean section (due to previous cesarean section) and gave birth to a female baby weighing 2,480 g. Apgar scores were 7 at 1 minute and 8 at 5 minutes, and the pH of umbilical artery blood was 7.327. The baby did not have any congenital anomalies, and the clinical course was uneventful. Twelve months have passed since the cesarean section, the patient has been free of disease, and the growth of the infant has been satisfactory.

## 3. Discussion

Esophageal cancer in pregnancy is extremely rare, because esophageal cancer occurs mainly in males in their sixties and seventies. There have been only five cases of esophageal cancer in pregnancy [4–8] thus far, as shown in Table 1. Four cases were primary esophageal cancer diagnosed in toon average, and the initial symptom was dysphagia in all cases. The prognosis of these cases was poor due to the aggressive nature of esophageal cancer. In the present case, however, recurrence was detected early as the localized disease by our regular trimonthly follow-up, and both maternal and fetal outcomes were favorable, while the median survival duration of the patients with recurrent disease after radical esophagectomy is 5 to 10 months [9].

When cancer is detected in pregnancy, especially before 22 weeks of gestation, both the patient and physicians are caught in a dilemma of whether to continue or terminate the pregnancy. In general, therapeutic strategies for cancer in pregnancy should adhere to the standard for nonpregnant patients, and treatment intensity should not be reduced to guard against potential adverse effects to the fetus. In the present case, because the patient and her husband strongly wished to continue the pregnancy, the multidisciplinary team assessed the feasibility of treatment during pregnancy. In order to permit the pregnancy continuation, it is essential to confirm no metastasis, and the patient underwent PET/CT. A full PET/CT scan is thought to deliver no greater than 15-20 mGy, and the placenta concentrates 0.19-0.27% of injected activity of 18F-FDG [10, 11]. In general, the dose of 18F-FDG and radiation is reduced as much as possible for pregnant women to lessen fetal exposure. Indeed, in the present case, the estimated maternal radiation dosage by PET/CT was calculated to be 3.8 mSv; PET 2.1 mSv, and CT 1.7 mSv, which was approximately equal to 3.8 mGy. Accordingly, PET/CT during pregnancy might be allowed, given that PET/CT plays a pivotal role in diagnosis and surveillance of metastatic lesions.

It is acknowledged that cancer chemotherapy is possible after 12 to 14 weeks of gestation, which is the period of organogenesis [12]. Cisplatin and fluorouracil are key drugs for chemotherapy in esophageal cancer [13, 14], and both have a relatively extensive history of use in pregnant patients from the second trimester onward. Mir et al. reported that, among 36 patients receiving cisplatin, one case of polyhydramnios, two cases of oligohydramnios, and three cases of fetal growth restriction were detected, although the causative link between cisplatin administration and these malformations remains speculative [15]. For 5-fluorouracil, one cohort study including 57 breast cancer patients receiving 5-fluorouracil during the second and third trimesters of pregnancy described three cases of congenital disease, including trisomy 21, club foot, and bilateral ureteral reflux [16]. The causal relationship between these diseases and 5-fluorouracil is unknown.

In our case, the newborn was relatively small at 2,480 g (13.2 percentile), although she was not regarded as small-for-gestational-age (SGA). It remains inconclusive whether cancer chemotherapy or cancer per se in pregnancy affects fetal growth. Cardonick et al. analyzed 376 fetuses exposed to chemotherapy* in utero* and reported that the incidence of fetal growth restriction was 7%, which might be as low as that in the normal population [17]. However, Van Calsteren et al. showed that SGA was observed significantly more often (16/66, 24.2%) in cancer patients given cytotoxic treatment [18]. Presumably, in the present case, CRT-induced pharyngitis and anorexia caused poor maternal weight gain during pregnancy, thereby contributing to the relatively light body weight of the newborn.

Meanwhile, radiotherapy during pregnancy could also be acceptable when the irradiation area is remote from the pelvis; several kinds of malignancies, such as breast cancer, Hodgkin's disease, brain tumors, and head and neck cancer, are treated with radiation therapy during pregnancy [19]. However, careful planning and calculation of the fetal dose using a phantom before treatment are necessary in order to assess the risk of radiation to the fetus. To evaluate and estimate the fetal dose, the Monte Carlo method can be employed. Bednarz and Xu demonstrated the computational procedure which could determine the absorbed organ dose in the mother and fetus, using realistic models of pregnant women during 3-, 6-, and 9-month gestational age [20]. In the current case, fetal dose was estimated at most 5.28 mGy even after 60 Gy in 30 fractions of radiotherapy. It was under the threshold dose for deterministic effects (50 mGy) regarding radiation injury to an unborn fetus [21]. Although the number of cases of chemotherapy and radiotherapy in pregnancy has been accumulating, CRT during pregnancy is still rare. To our knowledge, there has been no systematic review or cohort study verifying the adverse effects of CRT in pregnancy on the unborn fetus. Only in the fields of tongue cancer and Hodgkin's disease have a few cases of CRT in pregnancy been reported, and its influence on babies is currently unknown [22–24].

In summary, this is the first case of recurrent esophageal cancer in pregnancy in which CRT was completed without reducing treatment intensity and led to a complete response. Moreover, severe perinatal complications were not detected, and both the maternal and fetal courses were satisfactory. Our case suggests that CRT based on careful simulation could be an effective alternative treatment strategy for recurrent esophageal cancer in pregnancy. Nevertheless, little is known in terms of the safety and possible adverse effects of CRT on the fetus. Therefore, deliberate selection of patients and long-term follow-up of the child are necessary.

## Figures and Tables

**Table 1:** Cases of esophageal cancer during pregnancy reported in the literature.

Case	Age	Primary or recurrence	Timing of diagnosis (weeks of gestation)	symptoms	Treatment	Delivery (weeks of gestation)	Fetal outcome	Maternal outcome
Sharma et al. 2009	36	Primary	29	dysphagia, hematemesis	Surgery after delivery	31 CS	2,200 g uneventful	Death
Al-Githmi et al. 2009	29	Primary	29	dysphagia, weight loss	Surgery after delivery	32 CS	unknown	Death
Jain et al. 2014	27	Recurrence	26	dysphagia, weight loss	Chemotherapy during pregnancy	34 CS	2,300 g uneventful	Survival
Sahin et al. 2015	26	Primary	27	dysphagia, weight loss	Surgery during pregnancy	32 VD	uneventful	Survival
Akdemir et al. 2016	39	Primary	28	dysphagia, weight loss	Refusal of treatment	34 CS	2,650 g uneventful	Unknown
The present case	41	Recurrence	16	none	CRT during pregnancy	38 CS	2,480 g uneventful	Survival

CRT, concurrent chemoradiation therapy; CS, cesarean section; VD, vaginal delivery.
